# Dynamics of lymphocyte subpopulations during Legionnaires' disease

**DOI:** 10.1186/cc11700

**Published:** 2012-11-14

**Authors:** CPC de Jager, EFA Gemen, J de Jongh-Leuvenink, IBB Walsh, RJF Laheij, T van der Poll, PC Wever

**Affiliations:** 1 Jeroen Bosch Hospital, 's-Hertogenbosch, the Netherlands; 2 Academic Medical Center, Center of Infection and Immunity Amsterdam and Center of Experimental and Molecular Medicine, Amsterdam, the Netherlands

## Background

Absolute lymphocytopenia (lymphocyte count <1.0 × 10^9^/l) is recognized as an important hallmark of the immune response to severe infection and observed in patients with Legionnaires' disease (LD). Furthermore, LD is characterized by accumulation of activated T cells in the lungs. To explore the immune response in patients with LD, we studied the dynamics of peripheral blood lymphocyte subpopulations in the acute and subacute phase of the disease.

## Methods

EDTA-anticoagulated blood was obtained from eight LD patients on the day the diagnosis was made (acute phase) through detection of *Legionella pneumophila *serogroup 1 antigen in urine. A second blood sample was obtained in the subacute phase. Multiparametric flow cytometry was used to calculate absolute lymphocyte counts and B-cell, T-cell, NK-cell, CD4^+ ^and CD8^+ ^T-cell counts. Expression of activation markers was analyzed on CD4^+ ^and CD8^+ ^T cells. C-reactive protein (CRP) levels were used to monitor treatment response.

## Results

**Figure 1:**
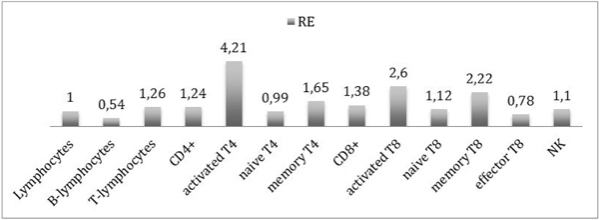
**Relative expansion (mean values) of lymphocyte subsets compared with the absolute lymphocyte count in the subacute phase compared with the acute phase of Legionnaires' disease**.

The absolute lymphocyte count (×10^9^/l, mean ± SD) significantly increased from 0.8 ± 0.4 to 1.7 ± 0.9 in the subacute phase. B-cell counts showed no significant change, while the T-cell count (×10^6^/l) significantly increased in the subacute phase (481 ± 283 vs. 1,290 ± 738) as a result of significant increases in both CD4^+ ^and CD8^+ ^T-cell counts (345 ± 168 vs. 898 ± 390 and 124 ± 104 vs. 333 ± 265). In the CD4^+ ^and CD8^+ ^T-cell populations, significant increases were observed in the subacute phase in absolute counts of activated CD38^+ ^HLA-DRA^+ ^cells (11 ± 7 vs. 81 ± 60 and 14 ± 13 vs. 68 ± 51) and CD45RA^- ^memory cells (141 ± 68 vs. 478 ± 185 and 28 ± 21 vs. 111 ± 44). Figure 1 shows the relative expansion (relative decrease/increase of a lymphocyte subset related to the relative increase of the absolute lymphocyte count) of the different lymphocyte subsets. The CRP level (mg/l) decreased from 359 ± 72 to 33 ± 17 in the subacute phase.

## Conclusion

The acute phase of LD is characterized by absolute lymphocytopenia which recovers in the subacute phase with an increase in absolute T-cell count and emergence of activated and memory-type CD4^+ ^and CD8^+ ^T cells. This confirms a role for T-cell activation in the immune response to LD.

